# Supplementary material for: Time-Dependent Molecular Motifs of Pulmonary Fibrogenesis in COVID-19
Source: Int J Mol Sci. 2022 Jan 29;23(3):1583. doi: 10.3390/ijms23031583 (PMC8835897; doi:10.3390/ijms23031583)
Supplement: Supplementary file 1 [file ijms-23-01583-s001.zip › Document S1.pdf]

Document S1. Information on further significantly regulated genes.

In the late group, there was an increased expression of HLA-A and HLA-B. Human leukocyte antigens (HLA) play an important role in immune responses to infections as well as in the development of acquired immunity [Delves et al.]. These antigens are expressed in several human cells, particularly in monocytes and other inflammatory cells as well as in variant epithelial cells. The role of several subtypes such as HLA-A and HLA-B in COVID-19 has been studied by lots of groups. However, no reliable relationship between distinct HLA subtypes and disease severity or susceptibility could be shown. [De Marco et al.] + [Schetelig et al.] + [Tomita et al.] + [Gutiérrez-Bautista et al.] The increased expression signature of HLA-A and HLA-B in the late group of this study is most likely caused by the altered cellular composition due to the inflammatory and fibrosing processes.

Further genes that showed increased activity in the late group were ITGB1, MMP1/14, PGK1, SERPINH1, ADAM9, BAX, CKAP4, CSNK1E, and IGF1. Of those, ITGB1 and MMP14 were also analyzed using immunostaining. As depicted in figure C1, MMP14 and ITGB1 showed a significantly increased expression in the late group, corresponding with the mRNA expression results. In the early group, MMP14 was limited to the adventitial layer of the pulmonary artery branches whereas an extensive high-intensity staining of the alveolar septa and large areas of fibrotically re-modeled tissue appeared in the late group. Similarly, ITGB1 showed a considerably stronger enhancement in the late compared to the early group, predominantly in the in-ternal and external elastic lamina of the pulmonary artery branches as well as in large areas of connective tissue within the thickened alveolar septa. ITGB1 encodes a beta subunit of a member of the integrins, a family of membrane receptors involved in cell adhesion, cell recognition and many more biological processes. [Abraham et al.] MMP1 and MMP14 encode members of the matrix metalloproteinases. These proteins are involved in the physiological breakdown of extracellular matrix, i.e. in developmental and remodelling processes, as well as in certain diseases, e.g. arthritis. In addition, MMPs are involved in lung repair processes following acute lung injury. [Aschner et al.] PGK1 is a glycolytic enzyme related to carbohydrate metabolism by catalyzing the conversion of 1,3-diphosphoglycerate to 3-phosphoglycerate. PGK1 is a transcriptional target of HIF1A and up-regulation has been shown in critically ill COVID-19 patients in a recent work. [Taniguchi-Ponciano]

SERPINH1 is a collagen-specific molecular chaperone and involved in fibrosing processes as it contributes to collagen biosynthesis. ADAM9 is a further member of the ADAM family

involved in distinct cell-cell and cell-matrix interactions, particularly in several human cancer entities. [Lin et al.] + [Zigrino et al.] Moreover, Rinchai et al. postulated that ADAM9 may constitute a valuable biomarker for the assessment of inflammation-related tissue damage. [Rinchai et al.] BAX encodes a protein that forms a heterodimer with the mitochondrial membrane protein B-Cell CLL/Lymphoma type 2 (BCL2), and therefore functions as an apoptotic activator. [Er et al.] CKAP4 encodes a protein with several functions, e.g. in the innate immune system. It has been identified as a Dickkopf-1 (DKK1) receptor while binding promotes proliferation of normal cells and cancer cells. [Kimura et al.] Recently, elevated CKAP4 plasma levels have been described to be associated with disease severity in COVID-19 [Patel et al.] CSNK1E is a member of the casein kinase I protein family which is involved in distinct intracellular control processes including DNA replication and repair. [Cheong et al.] IGF-1 is a growth factor involved in vascular protection, tissue repair, and inhibiting pro-inflammatory cytokine production. [Munoz et al.] Increased activity of IGF-1 and its downstream signaling pathways PI3K/AKT and MAPK has been shown following influenza A virus infection and linked with the inflammatory response induction. [Li G et al.] It might be hypothesized that IGF-1 similarly participates in the inflammatory host response to SARS-CoV-2 infection like to influenza A infection.
