# Supplementary material for: Time-Dependent Molecular Motifs of Pulmonary Fibrogenesis in COVID-19
Source: Int J Mol Sci. 2022 Jan 29;23(3):1583. doi: 10.3390/ijms23031583 (PMC8835897; doi:10.3390/ijms23031583)
Supplement: Supplementary file 1 [file ijms-23-01583-s001.zip › ijms-1568072-supplemental final.pdf]

Table S1. Clinical information of the study group.

Patient #	Sex	Age (Years)	Day of Hospitalization at Death	Mechanical Ventilation	Hospital Site
**"Early group"**					
1	Male	68	3	No	Hannover
2	Male	96	3	Yes	Basel
3	Male	78	3	Yes	Basel
4	Male	74	3	No	Basel
5	Female	81	4	Yes	Basel
6	Male	80	7	Yes	Ulm
**Median (IQR 25–75)**		79 (75–80.75)	3 (3–3.75)		
**"Late group"**					
7	Male	74	12	Yes	Lüdenscheid
8	Female	83	12	Yes	Aachen
9	Male	63	14	Yes	Hannover
10	Male	91	14	Yes	Hannover
11	Female	68	14	N/A	Ulm
12	Male	54	21	Yes	Bremen
**Median (IQR 25–75)**		71 (64.25–80.75)	14 (12.5–14)		
**"Control group"**					
13–33	N/A *	N/A *			Hannover

#, number; * information not publicly accessible due to data protection regulations and respecting the anonymity of donors.

Table S2. Raw data/Results of the mRNA expression analysis.

Table S3. Increased activity of biological pathways.

Signaling Pathway	Week 1 vs. Controls		Week 2 and Later vs. Controls	
	Regulation	fdr	Regulation	fdr
ECM structure (NS)	**up**	**0.0008**	**up**	**0.0008**
Cell adhesion (NS)	**up**	**0.0008**	**up**	**0.0091**
Collagen Family (NS)	**up**	**0.0048**	**up**	**0.0008**
Epithelial to mesenchymal transition (NS)	**up**	**0.0048**	**up**	**0.0058**
ECM receptor interaction (NS)	**up**	**0.0062**	**up**	**0.0058**
Basement membrane (NS)	**up**	**0.005**	**up**	**0.0091**
Blood coagulation (NS)	**up**	**0.0171**	**up**	**0.1005**
Type I interferon signaling (NS)	**up**	**0.0283**	**up**	**0.1852**
MMP remodeling (NS)	**up**	**0.0300**	**up**	**0.0195**
MHC class I antigen presentation (NS)	**up**	**0.0300**	**up**	**0.3788**
Th 17 differentiation (NS)	**up**	**0.0300**	**up**	**0.5052**
Type II interferon signaling (NS)	**up**	**0.0323**	**up**	**0.4587**
Cell-ECM interaction (NS)	**up**	**0.0301**	**up**	**0.0010**
Cell proliferation (NS)	**up**	**0.0283**	**up**	**0.1413**
Cytokine signaling (NS)	**up**	**0.0405**	**up**	**0.5052**
NF-κB signaling (NS)	**up**	**0.0405**	**up**	**0.7046**
NLR signaling (NS)	**up**	**0.0442**	**down**	**0.6816**
Innate immune system (NS)	**up**	**0.0341**	**down**	**0.7232**
Type I interferon signaling pathway (GO)	**up**	**0.0433**	**up**	**0.1513**

Intrinsic apoptotic signaling pathway in response to endoplasmic reticulum stress (GO)	**up**	**0.0433**	**up**	**0.0076**
Collagen catabolic process (GO)	**up**	**0.3255**	**up**	**0.0076**
Collagen fibril organization (GO)	**up**	**0.7186**	**up**	**0.0113**
Extracellular matrix organization (GO)	**up**	**0.3415**	**up**	**0.0076**

NS, calculated using gene-pathway associations supplied by Nanostring; GO, calculated using the GeneOntology database; fdr, false discovery rate.

Table S4. More details on significantly regulated biological pathways.

Table S5. Semiquantitative analysis of the immunostaining results.

**Target of Antibody**	**Early Group**			**Late Group**			**Controls**		
**Compartment**	**ENC**	**EPC**	**ECM**	**ENC**	**EPC**	**ECM**	**ENC**	**EPC**	**ECM**
**MMP14**	-	(+)	+	+	++	++	+	++	+
**ITGB1**	++	++	-	+++	+++	+	+++	+++	+
**PDGFRB**	-	-	(+)	+	(+)	+	(+)	++	(+
**THBS2**	-	-	-	(+)	-	(+)	+	+	(+)
**Collagen I**	-	-	(+)	-	-	++	-	-	++
**Collagen III**	++	+	(+)	+++	+++	+++	-	-	(+)
**Collagen IV**	++	++	(+)	+++	+++	+	(+)	+	(+)
**Collagen V**	-	-	+++	+++	+++	+++	-	-	++
**Collagen VI**	-	+++	+++	-	+++	+++	-	-	+

ENC, endothelial cells; EPC, respiratory/alveolar epithelial cells; ECM, extracellular matrix structures; MMP14, matrix metalloproteinase 14; ITGB1, integrin beta type 1; PDGFRB, platelet derived growth factor receptor beta; THBS2, thrombospondin 2. +++, highly positive; ++, intermediate positive; +, slightly positive; (+), partially positive; -, negative.

Table S6. Overview of target antibodies used for immunohistochemistry.

**Target of Antibody**	**Product Name**	**Host Species**	**Pretreatment**	**Dilution**	**Manufacturer**
**MMP14**	Recombinant Anti-MMP14 antibody [EP1264Y] (ab51074)	Rabbit	Tris-EDTA buffer	1:50	Abcam
**ITGB1**	Recombinant Anti-Integrin beta 1 antibody [EPR1040Y] (ab134179)	Rabbit	Tris-EDTA buffer	1:200	Abcam
**PDGFRB**	Anti-PDGFR beta antibody [42G12] (ab69506)	Mouse	Tris-EDTA buffer	1:100	Abcam
**THBS2**	Anti-Thrombospondin 2 antibody (ab112543)	Rabbit	Tris-EDTA buffer	1:25	Abcam
**Collagen I**	Anti-Collagen I antibody [3G3]—BSA and Azide free (ab88147)	Mouse	Tris-EDTA buffer	1:25	Abcam
**Collagen III**	Monoclonal Antibody to Collagen Type III—Aff. Purified—AF5810	Mouse	Tris-EDTA buffer	1:400	Acris
**Collagen IV**	Anti-Collagen IV antibody (ab6586)	Rabbit	Pronase	1:50	Abcam
**Collagen V**	Anti-Collagen V antibody (ab7046)	Rabbit	Tris-EDTA buffer	1:100	Abcam
**Collagen VI**	Recombinant Anti-Collagen VI antibody [EPR17072] (ab182744)	Rabbit	Tris-EDTA buffer	1:200	Abcam

MMP14, matrix metalloproteinase 14; ITGB1, integrin beta type 1; PDGFRB, platelet derived growth factor receptor beta; THBS2, thrombospondin 2.

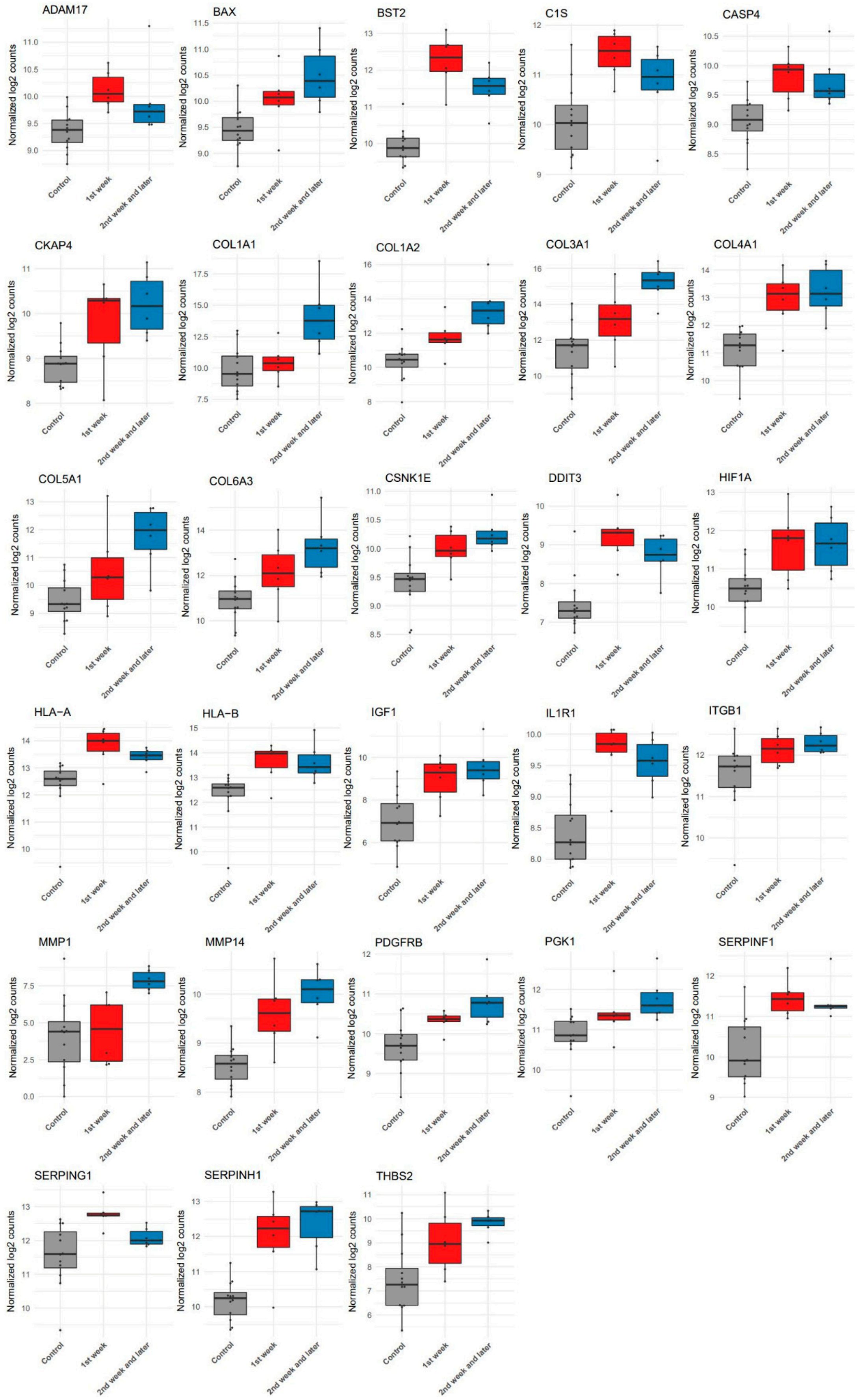

Figure S1. Box plots. Box plots of all genes with significantly increased mRNA expression.

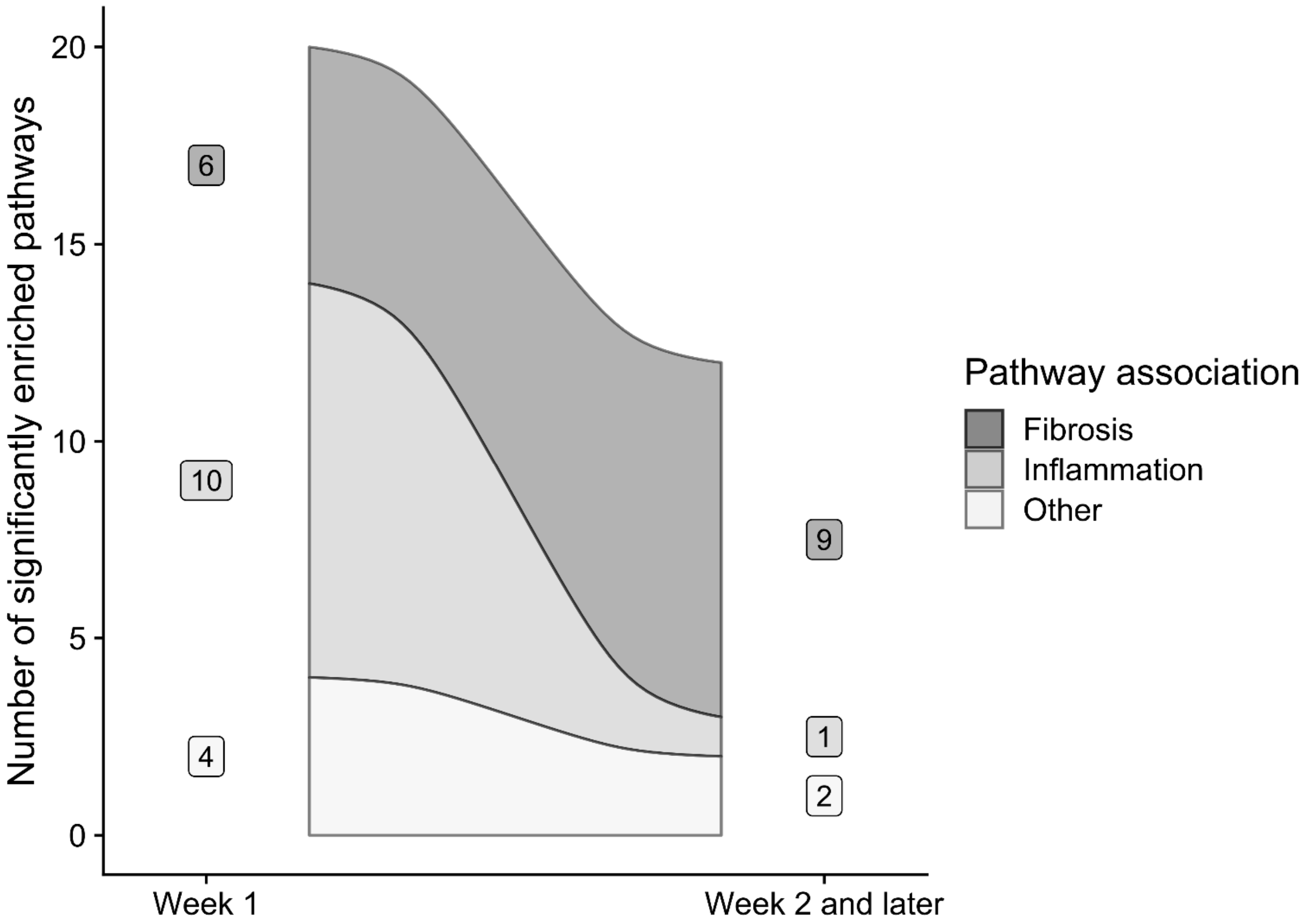

Figure S2. Flow plot. Flow plot illustrating the shift from inflammation-related to fibrosis-related pathways. Details on the association of pathways to inflammation and fibrosis are compiled in the supplementary Table S4.

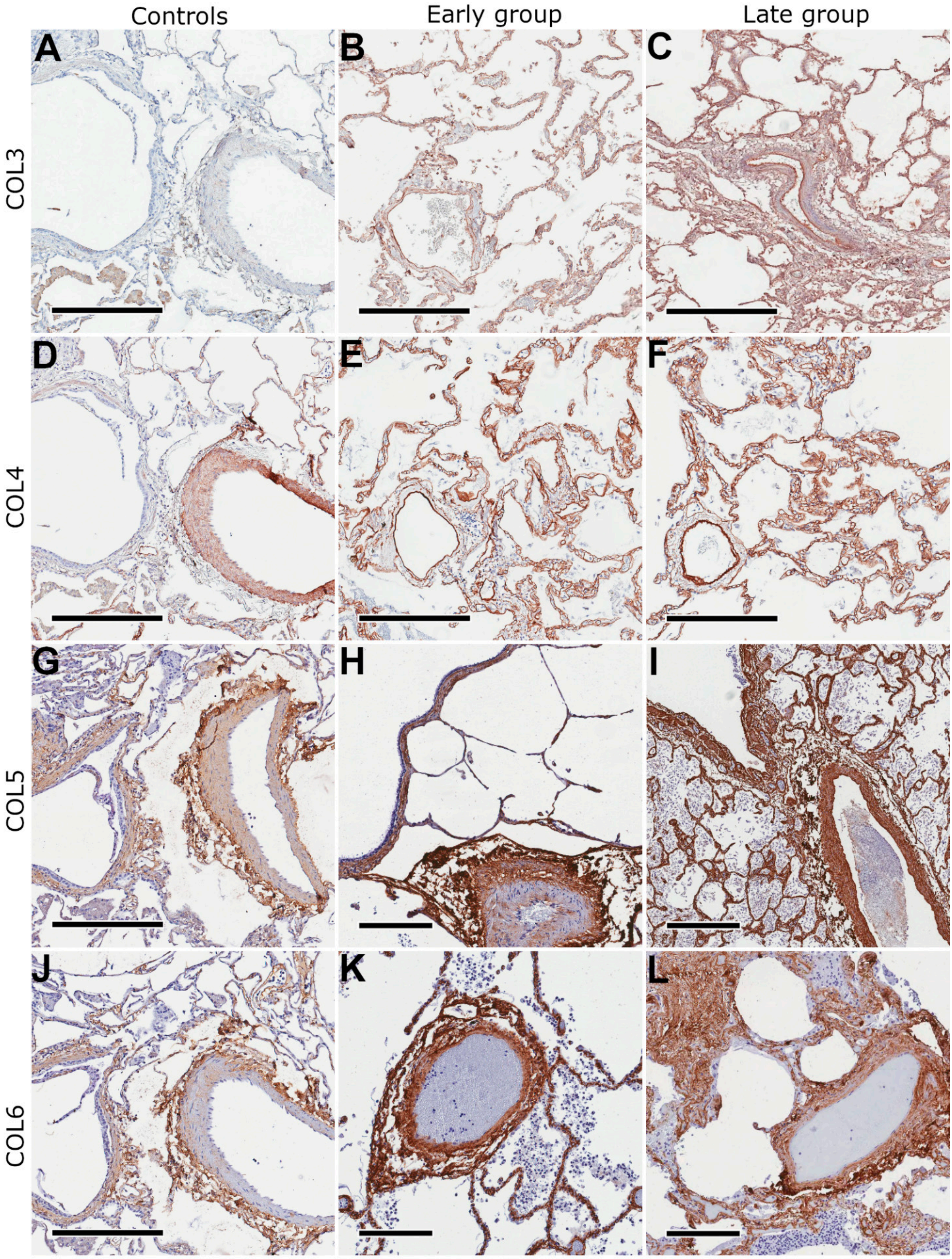

Figure S3. Immunostaining of collagens. Immunohistochemical appearance of several collagen subtypes in healthy control lungs, lungs from patients who succumbed to coronavirus disease 2019 (COVID-19) during the first week of hospitalization (early group) and lungs from patients who deceased at least 10 days after hospital admission (late group). In controls,

collagen 3 (COL3) (**A**) and collagen 4 (COL4) showed a slight enhancement within the bronchiolar wall, the media layer of the pulmonary artery and the alveolar basement membranes (**A**); COVID-lungs showed the same staining specificity for COL3 with a progression in size of stained structures in the early group (**B**) and again in the late group (**C**); COVID-19 lungs showed an enhancement of COL4 predominantly within the internal elastic membrane of pulmonary artery branches and the alveolar basement membrane; in controls, collagen 5 (COL5, (**G**) and collagen 6 (COL 6, (**J**) were enhanced predominantly within the perivascular stromal tissue and slightly also within the alveolar septa; in COVID-19 lungs, the staining intensity of both markers appeared higher due to a progression in size of COL5+ (**H**,**I**) and COL6+ (**K**,**L**) structures corresponding with progressive fibrotic remodeling; compared to the autopsy samples, staining appeared more intensive in healthy controls (**C**,**F**,**I**), presumably due to the higher tissue integrity; scale bares in (**A**–**G**, **J**) equal 300 μm; scale bars in (**H**,**K**,**L**) equal 200 μm; scale bar in (**I**) equals 500 μm.
